# Supplementary figures and images for: Abnormal platelet immunophenotypes and percentage of giant platelets in myelodysplastic syndrome: A pilot study
Source: PLoS One. 2022 Nov 21;17(11):e0278040. doi: 10.1371/journal.pone.0278040 (PMC9678267; doi:10.1371/journal.pone.0278040)

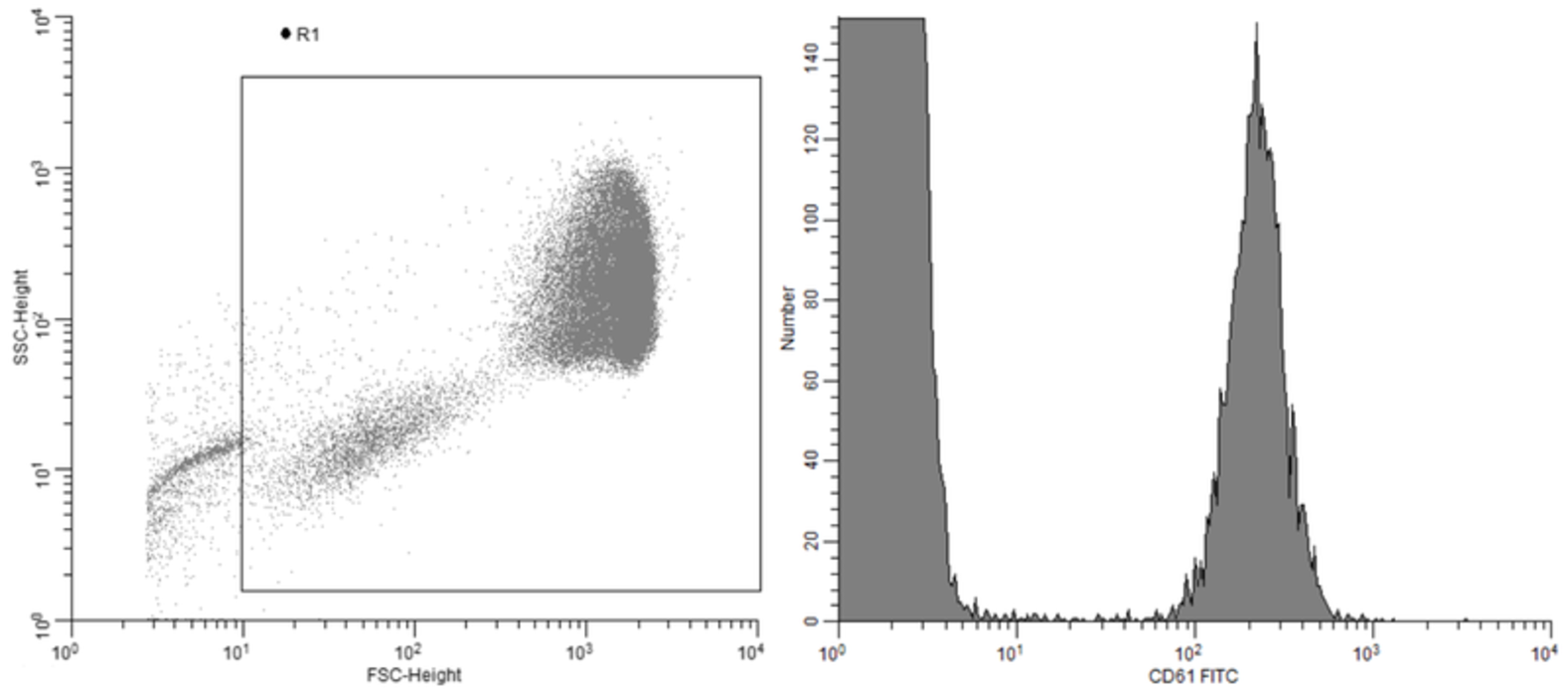

Supplement: S1 Fig — (A) A light scatter profile (FSC vs SSC) of diluted whole blood, in which blood cells are indicated in the rectangular region R1 (B) A histogram showing the blood cells with CD61 positivity. (TIF) [file pone.0278040.s001.tif]

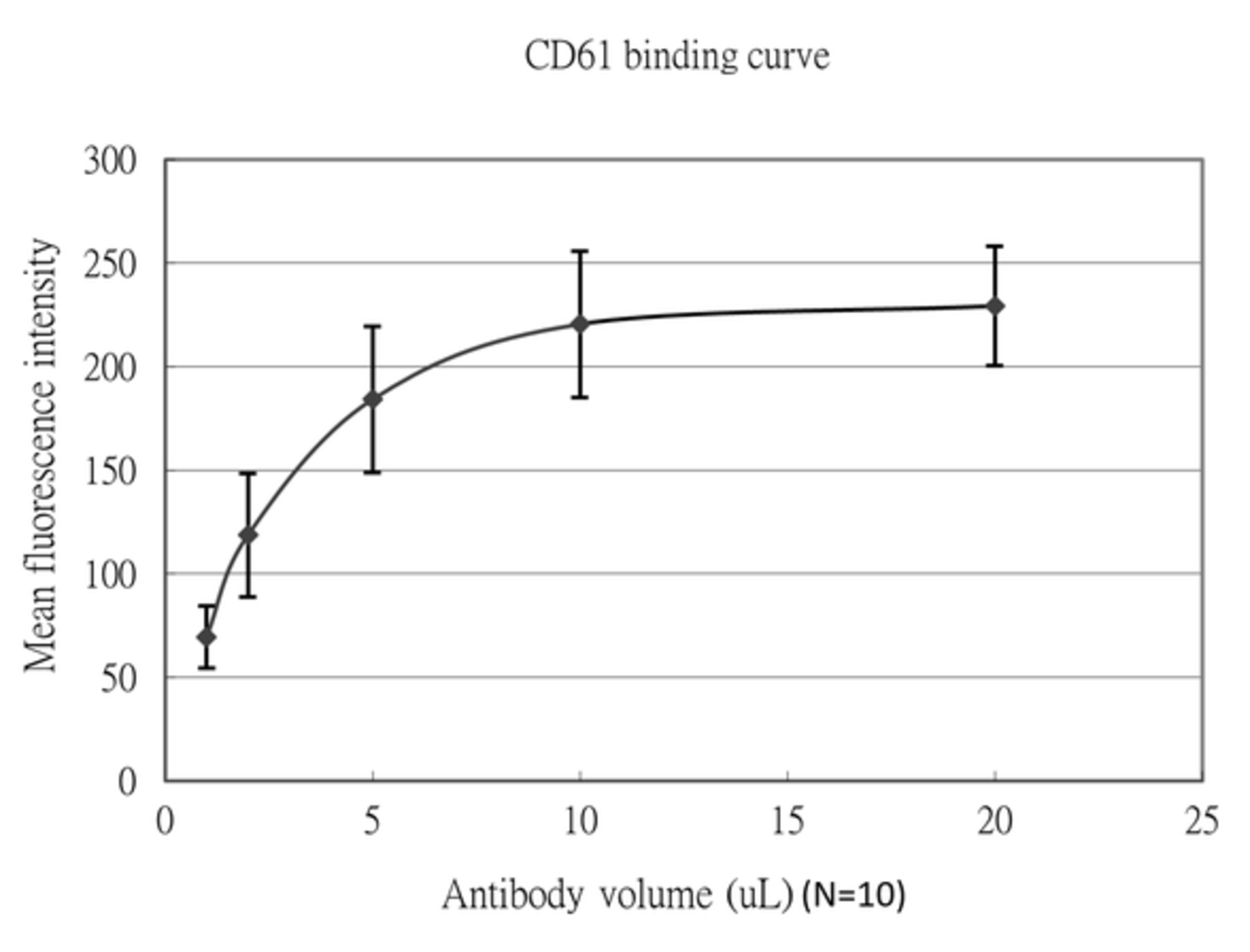

Supplement: S2 Fig — Data were shown in mean±SD (n = 10). (TIF) [file pone.0278040.s002.tif]
