# Supplementary material for: Abnormal platelet immunophenotypes and percentage of giant platelets in myelodysplastic syndrome: A pilot study
Source: PLoS One. 2022 Nov 21;17(11):e0278040. doi: 10.1371/journal.pone.0278040 (PMC9678267; doi:10.1371/journal.pone.0278040)
Supplement: Attachment — Submitted filename: Response to Reviewers R3.docx [file pone.0278040.s003.docx]

**Rebuttal letter (Response to Reviewers)**

**Response to Comments from the Editor and Reviewers (PONE-D-22-10816)**

Title: Abnormal platelet immunophenotypes and percentage of giant platelets in myelodysplastic syndrome: a pilot study

**Dear Editor and Reviewers,**

We thank the editor and reviewers for the extensive assessment of the manuscript, and the important and helpful comments and suggestions. We have taken all the remarks into account as described in detail below. We responded to all the comments and revised the manuscript accordingly. We wish the manuscript has improved considerably and suitable for consideration of publication.

**Reviewer#1**

1. The difference of size and platelet antigen expression by sex should be noted among MDS and healthy control group.

**Response:**

**We thank the reviewer’s comment. The distribution of sex between MDS groups and healthy controls was different. In MDS patients, the male: female was 15:5, and the healthy control was 8:12. The chi-square statistic is 5.0128. The p-value is 0.025. Above data was added in Table 1.**

**Although the sex between groups were significant different, we tried to analyze the size and platelet antigen expression by sex in healthy controls. Nevertheless, there were only twenty data for analysis, no significant differences were noted between male and female in percentage of giant platelet, FSC and antigen presentations.** **We added these results in discussion section.**

**In our results, under comparing between male and female, the P-value was 0.21 in percentage of giant platelets, 0.28 in FSC, 0.11~0.94 in antigen presentations with MFI, 0.31~0.85 in MFI adjusted by diameter. Under the published data by Oliver Heidmann Pedersen et al., no difference in platelet aggregation was found between sex and different age groups in a cohort of 171 Healthy Individuals with 87 men and 84 women and aged with 21–65years. Whether there are purely age-related changes in platelet surface markers is unclear.**

**(Reference: Cytometry B Clin Cytom. 2018 Nov;94(6):918-927. Platelet characteristics in patients with essential thrombocytosis)**

2. Density is calculated by mass / volume. The “density” in this article was defined as MFI / FSC ratio which is actually signal value / diameter. The term used in this article “density” might be changed to other terminology, such as signal to diameter ratio or MFI adjusted by diameter, etc.

**Response:**

**Thanks for reviewer’s suggestion. Indeed, the “density” is confused with the definition about MFI/FSC ratio. We had changed it to “MFI adjusted by diameter”.**

3. What was the relationship between dysmorphism of megakaryocyte in bone marrow aspiration or biopsy and platelet size or antigen density at the time of blood collection or flow cytometric analysis?

**Response:**

**From the medical records of bone marrow smear, all patients had dysplastic changing over megakaryocytes, including micro-megakaryocytes and separate nucleus. By the bone marrow smear, we only could separate to three groups with megakaryocytes’ numbers, including 10 patients with decreasing, 6 patients with increasing, and 4 patients with adequate megakaryocytes’ numbers. No significant differences were noted between three groups in percentage of giant platelet, FSC and antigen presentations. We added these results in results and discussion section.**

4. Was platelet size or antigen density related with bleeding tendency or thrombus generation?

**Response:**

**From the medical records, the symptoms of bleeding, including petechiae, ecchymosis, nasal bleeding, and gum bleeding while the patients were enrolled in this study, were found in 5 MDS patients who all had platelet counts less than 10,000/μL. No thrombosis events were noted in all patients. We analyzed the results between 5 patients with bleeding tendency and other 15 patients. No significant differences were noted between two groups in percentage of giant platelet, FSC and antigen presentations. We added these results in results and discussion section.**

**The P-value between the patient with and without bleeding tendency was 0.11 in percentage of giant platelets, 0.39 in FSC, 0.10~0.59 in antigen presentations with MFI, 0.11~0.51 in MFI adjusted by diameter. According to our data, the bleeding tendency may only correlate with platelet counts.**

5. Was platelet size or antigen density related with survival of patients?

**Response:**

**We tried to analyze the correlation between survival days after diagnosis and platelet size or antigen presentations. Because of different treatment for MDS, including supportive care only, Azacitidine, or allogenous transplantation, the survival days were heterogeneous. The average of survival days was 715.85 days. Until December 31st, 2021, only one patient still is survival (in high-risk group, post-allogenous transplantation, still complete remission). The correlation (R) with survival days was only 0.0658 with percentage of giant platelets, 0.0609 with FSC, 0.0248 with CD61 MFI, and 0.0009 with CD61 MFI adjusted by diameter. We added these results in discussion section.**

6. Were patients transfused with platelet product before blood drawn?

**Response:**

**As the mentions of methods, “peripheral blood was taken from MDS patients before receiving treatment.” All the patients did not receive platelets transfusion before the blood drawing for flow cytometry.**

7. Was platelet size or antigen density affected transfusion frequency?

**Response:**

**We collected the transfusion frequency from medical record. Because of the study is only performed by observation. All transfusion was given according to physician’s decision, and when platelet count is less than 20,000/μL, prophylactic transfusion with platelets was arranged. No correlation was noted between the platelet size and antigen presentations adjusted by diameter. The data was similar with bleeding tendency conditions.**

8. Is additional platelet related hematologic parameters available such as PDW, P-LCR, PCT that is provided by XE-5000?

**Response:**

**Although XE-5000 could supply the data of and PDW, P-LCR, PCT, if MPV was not available, above data also was not gotten from the XE-5000. In our data, there were 13 patients without MPV values. The data of MPV (fL) is calculated by PCT (platelet-crit)/PLT (platelet)*10000. Both PCT and PLT were measured by impedance method on Sysmex XN instruments. If fragment RBC or giant PLT were detected in specimens, the measurement of PCT would be interfered and the results were not available. Therefore, the data of MPV in these specimens cannot be provided.**

9. Giant platelets in peripheral blood are known to be present in high turnover state (ITP, MPN, MDS….) and falsely decrease platelet count. How was platelet count and ratio of giant platelet associated?

**Response:**

**We try to analyze the correlation between platelet counts and percentage of giant platelets in MDS patients. The R2=0.2328. In our data, no correlation was found between platelet counts and percentage of giant platelets because of small size of patients. We had added this results in discussion section.**

10. What is the clinical significance of measuring giant platelet, which is already known to be associated with high turnover state and of measuring antigen density?

**Response:**

**We thank for reviewer’s commend about platelet turnover. We tried to find the reference about turnover about platelets, but almost data was found in chronic myeloproliferative neoplasm with essential thrombocytosis and immune thrombocytopenia. There were few results in myelodysplastic syndrome. According to recent published by Oliver Buchhave Pedersen et al., the authors enrolled 47 patients with essential thrombocythaemia, and platelet function was evaluated by flow cytometry. Platelet turnover was assessed by immature platelet count, immature platelet fraction (IPF) and mean platelet volume (MPV). The patients have an accelerated platelet turnover leading to a higher proportion of newly produced immature platelets, and they also had significantly higher expression of platelet granule makers, P-selectin and CD63.**

**(Reference: Br J Haematol 2022 Jun 8. Cytoreductive treatment and association with platelet function and maturity in patients with essential thrombocythaemia.**

**Blood. 2012; 119(15): 3595– 603. Aspirin-insensitive thromboxane biosynthesis in essential thrombocythemia is explained by accelerated renewal of the drug target.)**

**In another study, platelet function and platelet turnover were increased in essential thrombocytosis patients compared with healthy controls, which CD36 and CD62P was increased.**

**(Reference: Semin Thromb Hemost. 2021 Feb;47(1):90-101. Platelet Function and Turnover in Essential Thrombocythemia: A Systematic Review.)**

**Form one review article, large platelets contain higher absolute amounts of proteins, including fibrinogen, glycoprotein Ia/IIa and IIb/IIIa complexes, glycoprotein VI, and the P2Y12 receptor. Although large platelets express 30 to 50% more glycoprotein (GP) Ia, GPIb, GPIIIa, GPVI and P2Y12 on their membranes compared with small platelets, functional differences of large and small platelets remain poorly understood because of a lack of standardized protocols separating platelets with different size.**

**(Reference: J Thromb Haemost. 2020 Jun;18(6):1256-1267. Large and small platelets-(When) do they differ?**

**Thromb Haemost. 2019 Mar;119(3):407-420. Role of Platelet Size Revisited-Function and Protein Composition of Large and Small Platelets.)**

**There was only one study which using autologous 111In-labeled platelets for platelet kinetics and the sites of platelet destruction in five thrombocytopenic patients with myelodysplastic syndromes. The patients with myelodysplastic syndromes had a normal pattern of platelet destruction, and recovery and survival day were similar between normal subjects and myelodysplastic patients.**

**(Reference: J Nucl Med. 1989 May;30(5):629-37. Platelet destruction in autoimmune thrombocytopenic purpura: kinetics and clearance of indium-111-labeled autologous platelets.)**

**By above study, higher turnover rate of platelets may have larger platelets and more glycoprotein receptors or antigen presentations. In our study, for the platelets of myelodysplastic syndrome, giant platelets and lower antigen presentations were found.**

**As above references and results, we had added in discussion section for platelet turnover.**

**Reviewer#2**

1. The authors have studied platelets in blood samples from 20 patients with myelodysplastic syndrome and 20 healthy controls collected in 2012. They aimed to compare the density of antigen expression of platelet associated CD31, CD36, CD41a, CD42a, CD42b and CD61 between these two groups using standard flow cytometry. Platelets were identified by light scatter (forward; side) and then assessed for expression of the specific molecule. There are a number of issues to be addressed to validate the findings and before the authors claim that this work “may help the diagnose MDS” [sic] can be sustained. These include technical and methodological.

There are discrepancies between the conclusions drawn in different parts of the manuscript. In the Abstract they conclude that in myelodysplasia there is lower antigen density of CD31, CD41a, CD42a, CD42b and CD61 on platelets. In the Results they state there is higher CD31 and CD36 antigen density, lower CD61 and no difference between MDS and normal for CD41a, CD42a and CD42b. These needs explaining.

**Response:**

**We are sorry for abstract which is not clear. We had corrected the abstract in revision manuscript. The MFI showed higher CD31 and CD36 antigen density, lower CD61 and no difference between MDS and normal for CD41a, CD42a and CD42b, but the antigen density (Corrected to “MFI adjusted by diameter”) was lower for CD31, CD41a, CD42a, CD42b and CD61 in MDS patients.**

**“Objectives: Myelodysplastic syndrome (MDS) is a heterogeneous hematopoietic stem cell disorder with thrombocytopenia. Flow cytometric immunophenotyping of blood cells has been instrumental in diagnosis as co-criteria, but the data regarding platelets remains lacking. This study aims to determine the level of antigen on platelets and to see if there is a difference between MDS patients and healthy controls, and giant platelet percentage between high- and low-risk MDS patients. Study design: Twenty newly diagnosed MDS patients were enrolled for this study. The antigen level on platelets was determined by measuring the binding capacity of antibody with flow cytometry. Results: Platelets of MDS patients were shown a lower level of CD61 and higher levels of CD31 and CD36 than healthy controls. Judged by forward scatter (FSC), MDS patients’ platelets appeared to be larger than healthy controls, whereas the MFI adjusted by diameter (MFI/FSC ratio) of CD31, CD41a, CD42a, CD42b and CD61 on platelets were lower in MDS patients than healthy controls. There was a significant quantity of giant platelets found in MDS patients, and the high-risk MDS patients tended to have a higher percentage of giant platelets than low-risk patients. Conclusions: All the results indicate that MDS patients exhibit a lower antigen presentation (MFI) adjusted by diameter on platelets than healthy controls and the giant platelets detected by flow cytometry might correlate with the condition of MDS.”**

2. Patients: Samples were from 20 patients with MDS, and the WHO 2006 classification was used. The authors should update these to WHO 2017. Why were indolent types of MDS not included (e.g. single lineage dysplasia). The blood count units should be in international units.

**Response:**

**We thank the reviewer’s commend for WHO 2006 and 2017. Because this study was performed before 2017, initially, we used WHO 2006 classification. We tried to publish our data recently, and the classification was updated to WHO 2017. We had updated the classifications of MDS to WHO 2017.**

**We only enrolled twenty MDS patients. There were no patients with single lineage dysplasia because no suitable patients with indolent types were enrolled during study period.**

**We also corrected the blood count units to international units. We had changed white blood count to *10^9^/L, and platelet count to *10^9^/L.**

3. The controls were not age-matched. What changes occur in the density of the antigens studied with normal healthy ageing?

**Response:**

**Data on the platelet surface markers of different age groups are limited. Huskens et al. assessed the effect of age on platelet activation in healthy volunteers. Results showed weak but significant correlations between αIIbβ3 receptor activation and response to TRAP and ADP and P-selectin expression and response to ADP. Under the published data by Oliver Heidmann Pedersen et al., no difference in platelet aggregation was found between sex and different age groups in a cohort of 171 Healthy Individuals with 87 men and 84 women and aged with 21–65years. Whether there are purely age-related changes in platelet surface markers is unclear.**

**(Reference: PLoS One 13(2) (2018) e0192079. Standardization and reference ranges for whole blood platelet function measurements using a flow cytometric platelet activation test.**

**Cytometry B Clin Cytom. 2018 Nov;94(6):918-927. Platelet characteristics in patients with essential thrombocytosis.)**

4. MDS patients had blast cells in their bone marrow (5.4 +/- 8.2%) and healthy controls 0%. Did controls have bone marrow examinations?

**Response:**

**I am sorry for the description. The percentage of blast cells were counted from peripheral blood only here. So, the healthy controls showed no blast cells in peripheral blood.**

5. Table 1: “There were 13 patients without MPV values” Can this data be included as the size of the platelets is important for the conclusions drawn.

**Response:**

**The data of MPV (fL) is calculated by PCT (platelet-crit)/PLT (platelet)*10000. Both PCT and PLT were measured by impedance method on Sysmex XN instruments. If fragment RBC or giant PLT were detected in specimens, the measurement of PCT would be interfered and the results were not available. Therefore, the data of MPV in these specimens cannot be provided.**

6. Flow cytometry: What was the time between sample collection (in 2012) and antigenic assessment? How were the platelets stored prior to study? What was done to prevent platelet activation prior to analysis? What impact did platelet activation have on the results? The authors should refer to the technical and methodological aspects of flow cytometry (e.g. citing MD Linden).

**Response:**

**We thank for reviewer’s suggestions. We had tried to explain more detail and clear. The antigen assessment was performed in thirty minutes after blood drawing and collection (all done in 2012), and platelets were not stored. EDTA was employed as the anticoagulant because it is a strong divalent chelator capable of inhibiting platelet activation and aggregation, as well as platelet-leukocyte aggregation. Although citrate was suggested for anti-coagulants of platelet study, we used EDTA as stronger inhibitors for prevention from platelet activation prior to analysis.**

**“Blood sample preparation**

**After the patients were enrolled in this study, peripheral blood was taken from MDS patients before receiving treatment, including target therapy or transfusion. It was collected into EDTA-containing tubes by venipuncture and kept at room temperature before analysis. EDTA was employed as the anticoagulant because it is a strong divalent chelator capable of inhibiting platelet activation and aggregation, as well as platelet-leukocyte aggregation. Around 5 mL of whole blood was collected for complete blood count (CBC) and flow cytometric analysis.”**

**“Flow cytometry**

**After blood drawing, below study about flow cytometry was performed in thirty minutes, and the platelets was not stored. The level of surface antigen on platelets was determined by the binding capacity of antibody with flow cytometry.”**

7. Antigen density has been inferred from the mean fluorescence intensity and forward scatter (size). Can the authors provide data calculated data from a standard using antibody binding capacity of beads. CD36 antigen density was higher in MDS than normal (Table 2). Was this due to platelet activation (“stimulation”)? There was no difference between CD41a antigen density between MDS and normal. This is different from Liu et al (Ref. 28). Why?

**Response:**

**We thank the reviewer’s commend for platelet activation. As above explaining, we used the EDTA for anticoagulants because EDTA is a strong divalent chelator capable of inhibiting platelet activation and aggregation, as well as platelet-leukocyte aggregation. Although we could not avoid all platelet activations, EDTA may be stronger inhibitors than citrate as anticoagulants.**

**About CD36, although CD36 is expressed following platelet stimulation and plays a role in mediating platelet-platelet and platelet-monocyte cell adhesion as well as cell attachment to the matrix, the stage of megakaryocytes’ maturation is still unclear. We only could explain that increasing CD36 caused from abnormal megakaryocytes’ maturation. By previous published by Alex F. Sandes et al, the results showed 27% MDS patients had increasing expression of CD36.**

**(Reference: Haematologica. 2012 Jun; 97(6): 895–902. Altered immunophenotypic features of peripheral blood platelets in myelodysplastic syndromes.)**

**Thanks for reviewer’s commend, and we tried to explain the difference between previous and our study. For the different results about CD41a antigen expression compared with Liu et al (Ref. 28), some different methods were noted that may cause the different results. First, we used EDTA as anticoagulant for prevention from platelet activation and aggregation, as well as platelet-leukocyte aggregation. Although citrate was suggested for anti-coagulants of platelet study, we used EDTA as stronger inhibitors for prevention from platelet activation prior to analysis. Second, we used only one fluoresce in each tube for avoid interfering with each other and needs with adjusting compensation. Third, the antigen saturation was very important for MFI. Before our study, we re-done the saturation curve for all antibodies. As methods, the adequate platelet count was needed to dilute to 10 * 10^9^/L, and 45 μL diluted whole blood was added with 10 μL of fluorochrome-conjugated antibodies was enough. We had added this discussion in revised manuscript.**

8. Platelet size was measured by MPV (fL), um and Forward Scatter. How do these correlate? How was 7um determined to define “giant platelets” in “blood and BM”?

**Response:**

**As reference from Stefan Handtke, et al, MPV changes depending on the time after blood collection and the anticoagulant used for blood sampling. A large bias was also recorded for between-instrument variations. The forward scatter for platelet size was not also accurate because resting platelets are small discoid shapes, not round shapes. But due to available data, forward scatter was used for this study.**

**(Reference: J Thromb Haemost. 2020 Jun;18(6):1256-1267. Large and small platelets-(When) do they differ?)**

**As above and results section, although forward scatter was not good enough for platelet size, but it was available by our data. The giant platelets were defined by the diameter of more than 7 μm by reference from Latger-Cannard V, et al. (Reference: Methods Mol Biol. 2013;992:207-25. Platelet morphology analysis)**

9. How was the percent giant platelets established? How was the data presented in Figure 5 established?

**Response:**

**As results section, giant platelets were defined by FSC>1000. We gated all platelets and the platelets with FSC>1000. The percentage of giant platelets was calculated by FSC>1000 platelets counts / total platelets count (gated by flow cytometry). The figure 5 was compared the percentage of giant platelets counts between healthy controls and patients with low-risk and high-risk MDS.**

10. There are typographical errors (e.g. “powder”, “diagnose” in Discussion)

**Response:**

**We tried to correct the errors as possible as we can. Thanks for reviewer’s mentions.**

11. The majority of references are pre-2010 (and only one in 2020 or later).

**Response:**

**We added new references for revised manuscript because of the suggestions and corrections from reviewers’ suggestions.**

**# Cytometry B Clin Cytom. 2018 Nov;94(6):918-927. Platelet characteristics in patients with essential thrombocytosis.**

**# Br J Haematol 2022 Jun 8. Cytoreductive treatment and association with platelet function and maturity in patients with essential thrombocythaemia.**

**# Blood. 2012; 119(15): 3595– 603. Aspirin-insensitive thromboxane biosynthesis in essential thrombocythemia is explained by accelerated renewal of the drug target.**

**# Semin Thromb Hemost. 2021 Feb;47(1):90-101. Platelet Function and Turnover in Essential Thrombocythemia: A Systematic Review.**

**# J Thromb Haemost. 2020 Jun;18(6):1256-1267. Large and small platelets-(When) do they differ?**

**# Thromb Haemost. 2019 Mar;119(3):407-420. Role of Platelet Size Revisited-Function and Protein Composition of Large and Small Platelets.**

**# PLoS One 13(2) (2018) e0192079. Standardization and reference ranges for whole blood platelet function measurements using a flow cytometric platelet activation test.**

12. The Figures 1 and 2 do not add to the manuscript and could be omitted. Figure is of poor quality

**Response:**

**We thank the reviewer’s suggestion, and we shifted the Figure 1 and 2 to Supplement. The figures’ quality was increased to 1200dpi.**

**Reviewer#3**

1. The potential role of immunophenotyping in diagnosis of MDS is perhaps over-stated. In most cases it is not required and merely supportive of morphological and genetic findings.

**Response:**

**We thank the reviewer’s suggestion. As reviewer’s mention, morphology and genetic finding was very important. About morphology, larger size of platelets was mentioned by previous textbooks and papers, but morphology is difficult to quantitative. We tried to gate the platelet counts and FSC by flow cytometry for quantitative about giant platelets.**

**About genetic findings, we could get the information about chromosome results from MDS patients. The genetic finding (by NGS) is not available for all patients in our hospital.**

2. It is stated that platelets have the immunophenotype of megakaryocytes, but there is no citation. Is this proven, or merely assumed?

**Response:**

**The GP IIb/IIIa and GPIb were expressed from promegakaryocytes, megakaryocytes and then platelets. We added the reference with Platelets and Megakaryocytes: Volume 1: Functional Assays, Volume: 272, 309-322, Jul-01-2004. Thanks for reviewer’s mention; and Exp Hematol. 2022 Feb;106:31-39. Integrins and their role in megakaryocyte development and function.**

3. The study was performed 10 years ago and uses an outdated MDS classification system and does not refer to IPSS-R.

**Response:**

**This result was delayed for publication because of the small size of patients initially when we tried to submit. Until recently, the flow cytometry for platelet is still controversial for MDS patients. Although only twenty patients were enrolled in this study, we think that may be helpful for further research about the important roles of platelets. Second, we found the similar study published by Liu et al (Ref. 28). Some different methods were noted that may cause the different results. We tried to submit our data again.**

**We had revised the classification of MDS to WHO 2017.** **We also calculated the IPSS-R score for our patients. The IPSS-R score was correlated with low- and high-risk as our previous subgroups. Nine patients with high risk had IPSS-R score with 5.5-9, and 11 patients with low risk had IPSS-R score with 1-4.5. We had added this data to our results.**

4. In Methods, please state how the platelet count was determined using Sysmex (impedance or fluorescence).

**Response:**

**Platelet counts were measured by impedance method on Sysmex XN instruments.**

5. The age of healthy control donors was substantially lower than in MDS patients. Are there any data on whether the MFI of platelet markers varies with age?

**Response:**

**Data on the platelet surface markers of different age groups are limited. Huskens et al. assessed the effect of age on platelet activation in healthy volunteers. Results showed weak but significant correlations between αIIbβ3 receptor activation and response to TRAP and ADP and P-selectin expression and response to ADP. Under the published data by Oliver Heidmann Pedersen et al., no difference in platelet aggregation was found between sex and different age groups in a cohort of 171 Healthy Individuals with 87 men and 84 women and aged with 21–65years. Whether there are purely age-related changes in platelet surface markers is unclear.**

**(Reference: PLoS One 13(2) (2018) e0192079. Standardization and reference ranges for whole blood platelet function measurements using a flow cytometric platelet activation test.**

**Cytometry B Clin Cytom. 2018 Nov;94(6):918-927. Platelet characteristics in patients with essential thrombocytosis.)**

6. Large platelets are typically seen in thrombocytopenia, so is the presence of large platelets simply a reflection of the severity of thrombocytopenia?

**Response:**

**We try to analyze the correlation between platelet counts and percentage of giant platelets in MDS patients. The R2=0.2328. In our data, no correlation was found between platelet counts and percentage of giant platelets because of small size of patients. We had added this results in discussion section.**

7. The paragraph that follows Table 1 in Results contains no results - belongs in Introduction/Methods.

**Response:**

**Thanks for reviewer’s suggestions. We had changed this section to “methods”.**

8. One of the problems with platelets in flow cytometry is the risk of clumping, which can sometimes happen with EDTA. How can we be certain that the giant platelets are single platelets and not clumps? The Sysmex MPV did not differ between MDS and healthy controls, which seems to indicate that there is no confirmed difference in size.

**Response:**

**Thanks for reviewer’s commend. Platelet clumping is most important problems for platelets study under flowcytometry. EDTA was employed as the anticoagulant because it is a strong divalent chelator capable of inhibiting platelet activation and aggregation, as well as platelet-leukocyte aggregation. Although citrate was suggested for anti-coagulants of platelet study, we used EDTA as stronger inhibitors for prevention from platelet activation prior to analysis. And all the patients when diagnosis, the smear of peripheral blood were done for excluding pseudo-thrombocytopenia.**

**Both PCT and PLT were measured by impedance method on Sysmex XN instruments. If fragment RBC or giant PLT were detected in specimens, the measurement of PCT would be interfered and the results were not available. Therefore, the data of MPV in these specimens cannot be provided. In our data, there were 13 patients without MPV values. And Sysmex will exclude the large cells (giant platelets) which were misidentified as fragment RBC.**

9. The sample size is too small to compare subsets of MDS, so the absence of statistically significant differences probably reflects inadequate power.

**Response:**

**Thanks for reviewer’s commend. As discussion section, the limitation of our study included small groups of patients. This is only a pilot study, and we tried to find the difference of platelets between MDS patients and healthy control, and high- and low-risk MDS patients. We will enroll more patients to confirm these results.**

10. Ref #28 seems to have done the same study as the authors, and found no useful differences. Why did the authors repeat the study? Did this study have some advantage that made it worthwhile to re-examine the question?

**Response:**

**Thanks for reviewer’s commend, and we tried to explain the difference between previous and our study. For the different results about CD41a antigen expression compared with Liu et al (Ref. 28), some different methods were noted that may cause the different results. First, we used EDTA as anticoagulant for prevention from platelet activation and aggregation, as well as platelet-leukocyte aggregation. Although citrate was suggested for anti-coagulants of platelet study, we used EDTA as stronger inhibitors for prevention from platelet activation prior to analysis. Second, we used only one fluoresce in each tube for avoid interfering with each other and needs with adjusting compensation. Third, the antigen saturation was very important for MFI. Before our study, we re-done the saturation curve for all antibodies. As methods, the adequate platelet count was needed to dilute to 10 * 10^9^/L, and 45 μL diluted whole blood was added with 10 μL of fluorochrome-conjugated antibodies was enough. We had added this discussion in revised manuscript.**

**Thanks for the editor and reviewers about the extensive assessment of the manuscript, and the important and helpful comments and suggestions again.**

**Sincerely**

**Yi-feng Wu / Tso-fu Wang**

**July 17^th^, 2022**
