# Supplementary material for: Abnormal platelet immunophenotypes and percentage of giant platelets in myelodysplastic syndrome: A pilot study
Source: PLoS One. 2022 Nov 21;17(11):e0278040. doi: 10.1371/journal.pone.0278040 (PMC9678267; doi:10.1371/journal.pone.0278040)
Supplement: Attachment — Submitted filename: Response to Reviewers R2.docx [file pone.0278040.s004.docx]

**Rebuttal letter (Response to Reviewers)**

**Response to Comments from the Editor and Reviewers (PONE-D-22-10816R1)**

Title: Abnormal platelet immunophenotypes and percentage of giant platelets in myelodysplastic syndrome: a pilot study

**Dear Editor and Reviewers,**

We thank the editor and reviewers for the extensive assessment of the manuscript, and the important and helpful comments and suggestions. We have taken all the remarks into account as described in detail below. We responded to all the comments and revised the manuscript accordingly. We wish the manuscript has improved considerably and suitable for consideration of publication.

**Reviewer#1**

1. The authors answered all the comments but, additional corrections are required.
2. The manuscript requires correction and revision by professional English user.

**Response:**

**Thanks for reviewer’s suggestion, we had sent to English editing before submission, and the manuscript had been reviewed with two experts in English again for this revision. The details showed in “Revised Manuscript with Track Changes R2”.**

**Reviewer#3**

1. The authors have addressed most of my comments.
2. The Sysmex instrument has multiple ways to determine the platelet count (e.g. impedance/fluorescence). Please specify which method was used.
3. Can giant platelets be distinguished from red cell fragments simply by FSC>1000? Was any lineage marker used to confirm?
4. The paragraph on survival in the discussion could be omitted. The sample size is too small.

**Response:**

1. **Thanks for reviewer’s suggestion.**
2. **The method with PLT-O (fluorescence) of Sysmex instrument was used for this study. We had added the detail in “Complete blood count test” section as “The PLT-O (fluorescence) channel of Sysmex instrument was used for this study.”**
3. **We used the platelet specific marker of CD61 for gating platelets for the ratio of “FSC>1000“ which could distinguish from red cell fragments. We added the detail explaining in “Giant platelets in the peripheral blood of MDS patients and healthy controls” section as “On gating platelets by the surface marker of CD61, a subgroup of platelets bearing exceptionally large size (FSC>1000)…”.**
4. **We had deleted the paragraph about survival analysis in the discussion section.**

**Thanks for the editor and reviewers about the extensive assessment of the manuscript, and the important and helpful comments and suggestions again.**

**Sincerely**

**Yi-feng Wu / Tso-fu Wang**

**Nov 5th, 2022**
